# Two New Species of *Liuixalus* (Rhacophoridae, Anura): Evidence from Morphological and Molecular Analyses

**DOI:** 10.1371/journal.pone.0136134

**Published:** 2015-08-25

**Authors:** Shaobo Qin, Yunming Mo, Ke Jiang, Bo Cai, Feng Xie, Jianping Jiang, Robert W. Murphy, Jia-Tang Li, Yuezhao Wang

**Affiliations:** 1 Chengdu Institute of Biology, the Chinese Academy of Sciences, Chengdu, Sichuan, China; 2 Natural History Museum of Guangxi, Nanning, Guangxi, China; 3 Centre for Biodiversity and Conservation Biology, Royal Ontario Museum, Toronto, Ontario, Canada; 4 College of Life Sciences, Sichuan University, Chengdu, Sichuan, China; The National Orchid Conservation Center of China; The Orchid Conservation & Research Center of Shenzhen, CHINA

## Abstract

Due to small body sizes, superficial similarities in morphologies, and obscure activity behaviors, the phylogeny and taxonomy of species in the genus *Liuixalus* were very troublesome. Some species might comprise a complex of cryptic species. To investigate the species of group, we constructed the matrilineal genealogy of the genus using 16s rRNA mitochondrial DNA sequences. Analyses recovered six well supported matrilines that involved *L*. *romeri*, *L*. *ocellatus*, *L*. *hainanus*, *L*. *calcarius*, *Liuixalus shiwandashan* **sp. nov.** and *Liuixalus jinxiuensis* **sp. nov.**, though the historical relationships among them remained unresolved. Currently, *Liuixalus* included 4 species, distributed eastwards from northern Vietnam to Hong Kong, China. Based on genealogical and morphological distinctiveness, we described *Liuixalus jinxiuensis* **sp. nov.** from the type locality Mt. Dayao, Jinxiu, China and *Liuixalus shiwandashan* **sp. nov.** from the type locality Mt. Shiwanda, China. A combination of morphological measurements, genetic, bioacoustic and osteological analysis was served to diagnose the new taxa.

## Introduction

The genus *Liuixalus* was proposed in 2008 with type species *L*. *romeri* (Smith, 1953) based on its distinct molecular phylogenetic position [1], which was confirmed by Li *et al*. [2] and Yu *et al*. [3]. All previous studies suggested that *Liuixalus* was a sister clade to all other members in the subfamily Rhacophorinae [1–5]. A small body size, the absence of vomerine teeth, strongly reduced webbing and X-shape dark colored mark on the shoulder served to diagnose *Liuixalus* [6–9]. *Liuixalus romeri* was described from Hong Kong but also found to occur in southern Guangxi. *Liuixalus hainanus* and *L*. *ocellatus* occured at Mt. Diaoluo (Diaoloushan), Hainan, China and Mt. Wuzhi (Wuzhishan), Hainan, respectively. Recently, Milto *et al*. [9] described *L*. *calcarius* and suggested it was endemic to Cat Ba Island, Gulf of Tonkin, Vietnam. Shortly thereafter, Nguyen *et al*. [10] described the new species *L*. *catbaensis* from the same locality. Based on their similar morphologies, same distributions, and close publication dates, Frost (2015) suggested that *L*. *catbaensis* was a junior synonym of *L*. *calcarius*.

Based on fieldwork in Mt. Dayao in 2008 and Mt. Shiwanda in 2013, we collected several specimens of two unknown forms of *Liuixalus*. Located in central Guangxi, southern China, Mt. Dayao is the watershed of the Gui and Liu rivers. Lying in south of Guangxi, Mt. Shiwanda stretches for over 100 km and covers an area of 2600 km^2^. This rolling mountain chain consists of 72 peaks over 500 m and 21 peaks over 1000 m a.s.l. The ecosystem in Guangxi is diverse owing to its particular geographic location and complicated environment. Previous surveys identified about 166 species of mammals, 483 birds, and 1766 Marine animals in Guangxi [11].

Accurate taxonomies form the foundation of conservation. The phylogenetic relationship among the *Liuixalus* requires extensive sampling and robust diagnoses. In this study, we firstly construct matrilineal genealogy of the group by analyses of mitochondrial DNA (mtDNA). The geneology makes a framework for morphological assessments. The results indicate the taxonomy of the genus *Liuixalus* more clearly and the necessity to describe two new species *Liuixalus shiwandashan* **sp. nov.** and *Liuixalus jinxiuensis* **sp. nov.**. Bioacoustic and osteological analyses enrich the species descriptions in critical ways.

## Material and Methods

### Ethics Statement

This work was conducted with the permission of the Management Offices of the Mt. Dayao and Mt. Shiwanda Nature Reserve. All animal procedures were approved by the Animal Care and Use Committee of Chengdu Institute of Biology (permission number: 20140401).

### Species Sampling and Data Collection

Field work was conducted in April to May 2008 at Mt. Dayao, Guangxi, China, and April to May 2013 at Mt. Shiwanda, Guangxi, China (Table 1). Specimens were ethanol-fixed and deposited in Herpetological Museum of the Chengdu Institute of Biology, Chinese Academy of Sciences (CIB), and Guangxi Zhuang Autonomous Region Museum of Natural History (GXNM). Character data for comparison were taken from specimens and references [6–10].

**Table 1:** Species used in study. “--” represents the unknown information from Genbank. CIB, Chengdu Institute of Biology, the Chinese Academy of Sciences; KIZ, Kunming Institute of Zoology, the Chinese Academy of Sciences; SCUM, Sichuan University Museum.

Taxon	Specimen voucher No.	Locality	CenBank accession nos. 16s
*Buergeria japonica*	UMFS 5821	Taiwan, China	DQ283055
*Buergeria buergeri*	--	Ota River, Hiroshima perfection, Japan	AB127977
*Buergeria oxycephala*	SCUM 050267YJ	Hainan China	EU215524
*Rhacophorus rhodopus*	SCUM 060692L	Mengyang Jinghong China	EU215531
*Polypedates megacephalus*	SCUM LJT 73	Yaan Sichuan China	KF053220
*Philautus jinxiuensis*	KIZ 061210YP	Mt. Dayao Guangxi China	EU215525
*Rhacophorus moltrechti*	SCUM 061106L	Lianhuachi Taiwan China	EU215543
*Chiromantis hansenae*	KUHE:34136	Nong Khor, southeastern Siam	AB813161
*Kurixalus hainanus*	HNNU A1180	Mt. Diaoluo Hainan China	EU215548
*Liuixalus ocellatus*	--	China	GU120328
*Liuixalus ocellatus*	--	Mt. Diaoluo, Hainan, China	AB871414
*Liuixalus ocellatus*	HN 0806046	Mt. Wuzhi, Hainan, China	KC465829
*Liuixalus ocellatus*	--	Mt. Wuzhi, Hainan, China	AB871417
*Liuixalus ocellatus*	HN 0806045	Mt. Wuzhi, Hainan, China	GQ285672
*Liuixalus ocellatus*	--	Mt. Diaoluo, Hainan, China	AB871413
*Liuixalus ocellatus*	--	Mt. Wuzhi, Hainan, China	AB871419
*Liuixalus ocellatus*	--	Mt. Wuzhi, Hainan, China	AB871418
*Liuixalus ocellatus*	--	Mt. Wuzhi, Hainan, China	AB871416
*Liuixalus ocellatus*	--	Mt. Wuzhi, Hainan, China	AB871415
*Liuixalus jinxiuensis* **sp. nov.**	KIZ 060821245	Guangxi, China	EF564535
*Liuixalus jinxiuensis* **sp. nov.**	CIB 101060	Mt. Dayao Guangxi, China	KT192635*
*Liuixalus jinxiuensis* **sp. nov.**	KIZ 060821246	Guangxi, China	EF564536
*Liuixalus calcarius*	--	Cat Ba island, Gulf of Tonkin, Vietnam	AB871420
*Liuixalus hainanus*	HN 0806039	Yinggeling, Hainan, China	KC465827
*Liuixalus hainanus*	HN 0806040	Yinggeling, Hainan, China	KC465828
*Liuixalus hainanus*	SCUM 060401L	Diaoluoshan, Hainan, China	GQ285671
*Liuixalus hainanus*	LJT V15	Diaoluoshan, Hainan, China	KC465826
*Liuixalus romeri*	--	Hong Kong, China	AB871412
*Liuixalus romeri*	CIB 10LJT	Hong Kong, China	KT192638*
*Liuixalus romeri*	CIB 7LJT	Hong Kong, China	KT192637*
*Liuixalus romeri*	CIB 6LJT	Hong Kong, China	KT192636*
*Liuixalus shiwandashan* **sp. nov.**	KIZ 061205YP	Mt. Shiwanda, Guangxi, China	EU215528
*Liuixalus shiwandashan* **sp. nov.**	CIB 101061	Mt. Shiwanda, Guangxi, China	KT192633*
*Liuixalus shiwandashan* **sp. nov.**	CIB 101054	Mt. Shiwanda, Guangxi, China	KT192634*

* Sequences new to this study.

### Molecular Analysis

Taxonomic sampling included 34 sequences. For our sequencing, genomic DNA was extracted from either muscle, or live tissues using the standard phenol-chloroform extract protocol [12]. Primer sequences of Wilkinson *et al*. [13–14] were used to amplify and sequence a fragment that included 1467 aligned nucleotide positions encompassing from 12S to 16S ribosome RNA (rRNA) of the mtDNA genome. Double-stranded polymerase chain reaction (PCR) amplification was carried out using the following parameter: 94°C initial hot start (3 min), then 30 cycles of 94°C denaturation (1 min), 52°C annealing (1 min), and 72°C extension (2 min). Final extension at 72°C was conducted 10 min. PCR products were directly sequenced with an ABI 3730 automated DNA sequencer and in both directions. The resulting sequences were submitted to Blast searching [15] in GenBank to ensure the required sequence had been sequenced. The outgroup taxa (and GenBank Accession Nos.) included *Buergeria japonica* (DQ283055), *Kurixalus hainanus* (DQ283054), *Rhacophorus moltrechti* (DQ283080), *Polypedates megacephalus* (DQ283073), *Chiromantics hansenae* (AB813161), *Buergeria oxycephala* (AB813156), *Rhacophorus rhodopus* (AB813151), *Philautus jinxiuensis* (EU215525), and *Buergeria buergeri* (AB127977).

**Fig 1:**
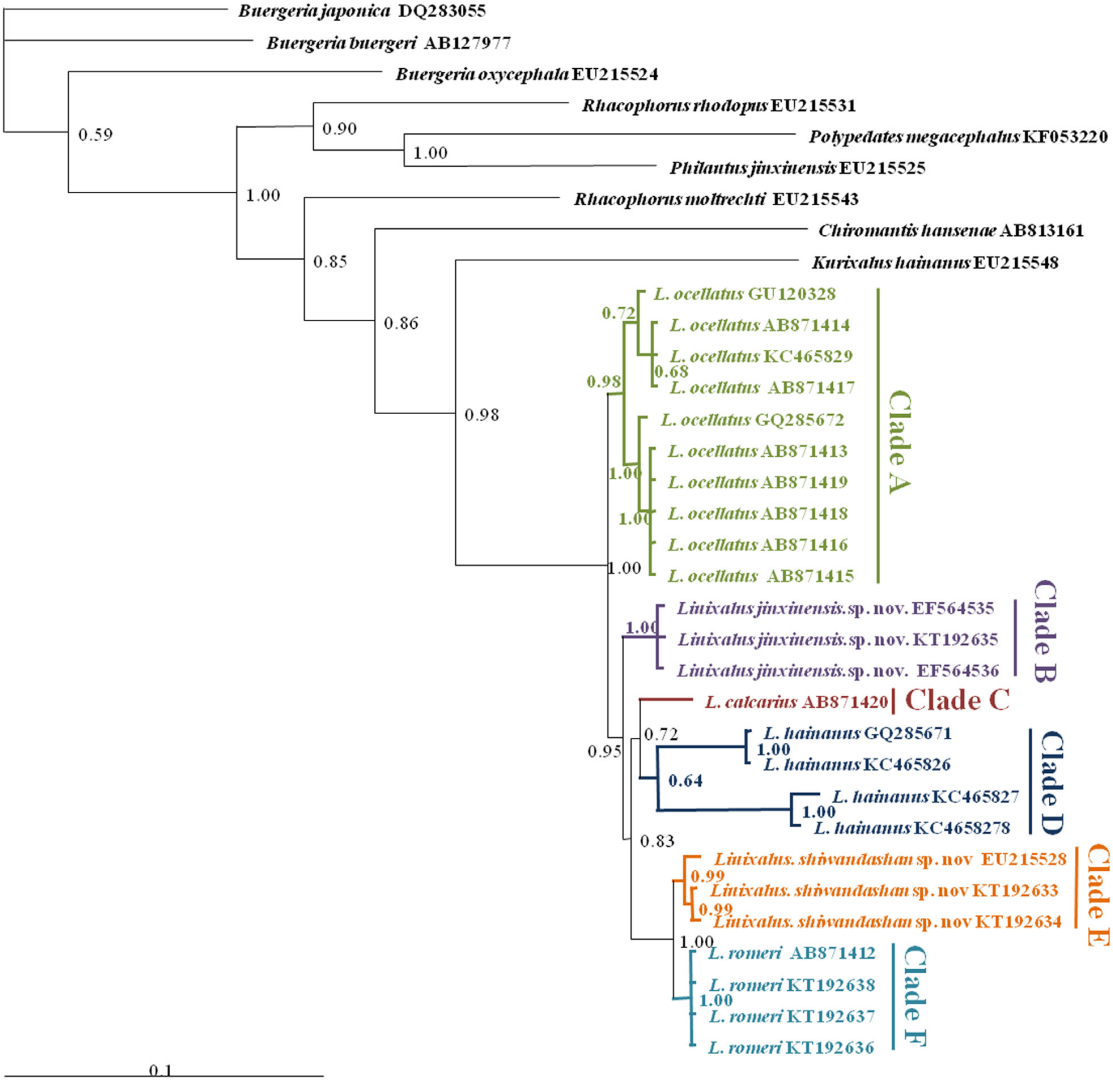
Bayesian inference tree. This tree was inferred from the mtDNA dataset. All of the Bayesian posterior probabilities (BPP) and NCBI numbers were shown.

Alignments first used CLUSTALX 1.81 [16–18] with default parameter followed by visual confirmation and manual adjustments. Nucleotide sites with ambiguous alignments were removed from analyses. Gaps were analyzed as missing data. Our *de novo* were trimmed to 16s rRNA only to match data downloaded from NCBI. The aligned sequences were analyzed using Bayesian inference (BI) in MrBayes 3.12 [19]. Markov Chain Monte Carlo (MCMC) generations used three million iterations and we sampled every 1000^th^ step. The first 25% of the samples were discarded as conservative burn-in. The remaining samples were used to generate a majority-rule consensus tree (Fig 1).

### Morphological Analysis

Measurements were taken with dial calipers with a precision of 0.1 mm under binocular dissecting microscope by one person to preclude variation owing to researcher-bias. The following measurements and abbreviations were used: SVL = snout-vent length; A-G = axilla to groin, distance from posterior base of forelimb at its emergence from body to anterior base of hind limb at its emergence from body; HW = head width at the greatest cranial width; HL = head length from the rear of the lower jaw to the tip of the snout; HD = head depth, greatest transverse depth of head, taken beyond interorbital region; UEW = upper eyelid width: greatest width of upper eyelids; IOD = interorbital distance; ED = horizontal diameter of eye; TD = horizontal diameter of tympanum; ESL = tip of snout-eye distance; TED = tympanum-eye distance from anterior edge of tympanum to posterior corner of the eye; IND = inter-narial distance: distance between nostrils; END = eye to nostril distance: distance from anterior corner of eye to nostril; FLL = length of forelimb from tip of disk of finger III to axilla; FFL = first finger length; TFL = third finger length; FTD = maximal diameter of disc of finger III; and NPL = nuptial pad length.

### Bioacoustics Analysis

Calls were collected at Mt. Shiwanda, Guangxi, China from April to May 2013 using a Nikon P310 recorder, and at our sampling locality Mt. Dayao from April to May 2008 using a Canon. The calls were frequently heard at 19:00–23:00. Spectrograms of male calls were generated using Avisoft-SAS lab lite software with a 22.05 kHz sampling frequency and 16-bit precision.

### Osteological Analysis

The skeletons of one adult male from each locality were analyzed as cleared and stained specimens prepared according to Wassersug’s [20–21] protocol. The terminology for cranial and postcranial osteology followed Trueb [22–23], Heyer [24] Lynch [25], and Trueb *et al*. [26].

### Nomenclatural Acts

The electronic edition of this article conforms to the requirements of the amended International Code of Zoological Nomenclature, and hence the new names contained herein are available under that Code from the electronic edition of this article. This published work and the nomenclatural acts it contains have been registered in ZooBank, the online registration system for the ICZN. The ZooBank LSIDs (Life Science Identifiers) can be resolved and the associated information viewed through any standard web browser by appending the LSID to the prefix “http://zoobank.org/”. The LSID for this publication is: urn:lsid:zoobank.org:pub: 38D7B0EB-90FF-4817-9579-E0C101119749. The electronic edition of this work was published in a journal with an ISSN, and has been archived and is available from the following digital repositories: PubMed Central, LOCKSS.

## Result

### Sequence Variation

The aligned rDNA gene fragments from *Liuixalus* consisted of 1467 nucleotide positions before trimming. The posterior 869 nucleotide positions were retained for genealogical reconstructions. The fragments contained 484 constant and 385 potentially phylogenetically informative characters. Plots of transitions and transversions showed a linear relationship, thus, giving no indication of saturation effects. Consequently, all nucleotide positions were used for genealogical inference.

### Phylogenetic Analysis

Likelihood value of the 50% majority consensus tree was lnL = -5885.820. The standard deviation of split frequencies among the four BI runs was 0.002966. The following relationships were indicated as being well supported and reliable:

1. Monophyly of *Liuixalus* was strongly supported (BPP = 1.00).
2. Matriline A included *L*. *ocellatus* from the different localities: Mt. Wuzhi, Hainan, Mt. Diaoluo, Hainan and a vague locality (BPP = 0.98). This lineage was the sister-group to all the other matrilines (B, C, D, E and F), as association that received moderate support (BPP = 0.95).
3. Matriline B, which contained *Liuixalus jinxiuensis* **sp.nov.** from Jinxiu, Guangxi, China, received strong nodal support (BPP = 1.00). It formed the sister-group to matrilines C, D, E, and F. Matriline C included *L*. *calcarius* from the type locality Cat Ba island, Gulf of Tonkin, Vietnam. Matriline D contained *L*. *hainanus* from the Mt. Diaoluo, Hainan and Yinggeling, Hainan. Matrilines C and D were moderately supported sister species (BPP = 0.72). Finally, strongly supported sister matrilines (BPP = 1.00) E and F contained *Liuixalus shiwandashan* **sp. nov.** from Mt. Shiwanda, Guangxi, China and *L*. *romeri* from the type locality Hong Kong, respectively.

The genealogy was assumed to represent historical relationship of the species, i.e. resolution of a paternal genealogy would yield the same six lineages. Given prior recognition of most taxa, the assumption was not rejected. Thus, the genealogy served to reject the H_0_ of conspecificity of all six matrilines due to evolutionary cohesiveness within the current taxonomy. Consequently, we were required to describe two new species so that the taxonomy replicated historical relationships.

## Species Accounts

### *Liuixalus shiwandashan* sp. nov.

Li, Mo, Jiang, Xie & Jiang

urn:lsid:zoobank.org:act:DD566B0F-1ABE-443A-A73E-FA9FB9EE957E

#### Holotype

**Fig 2:**
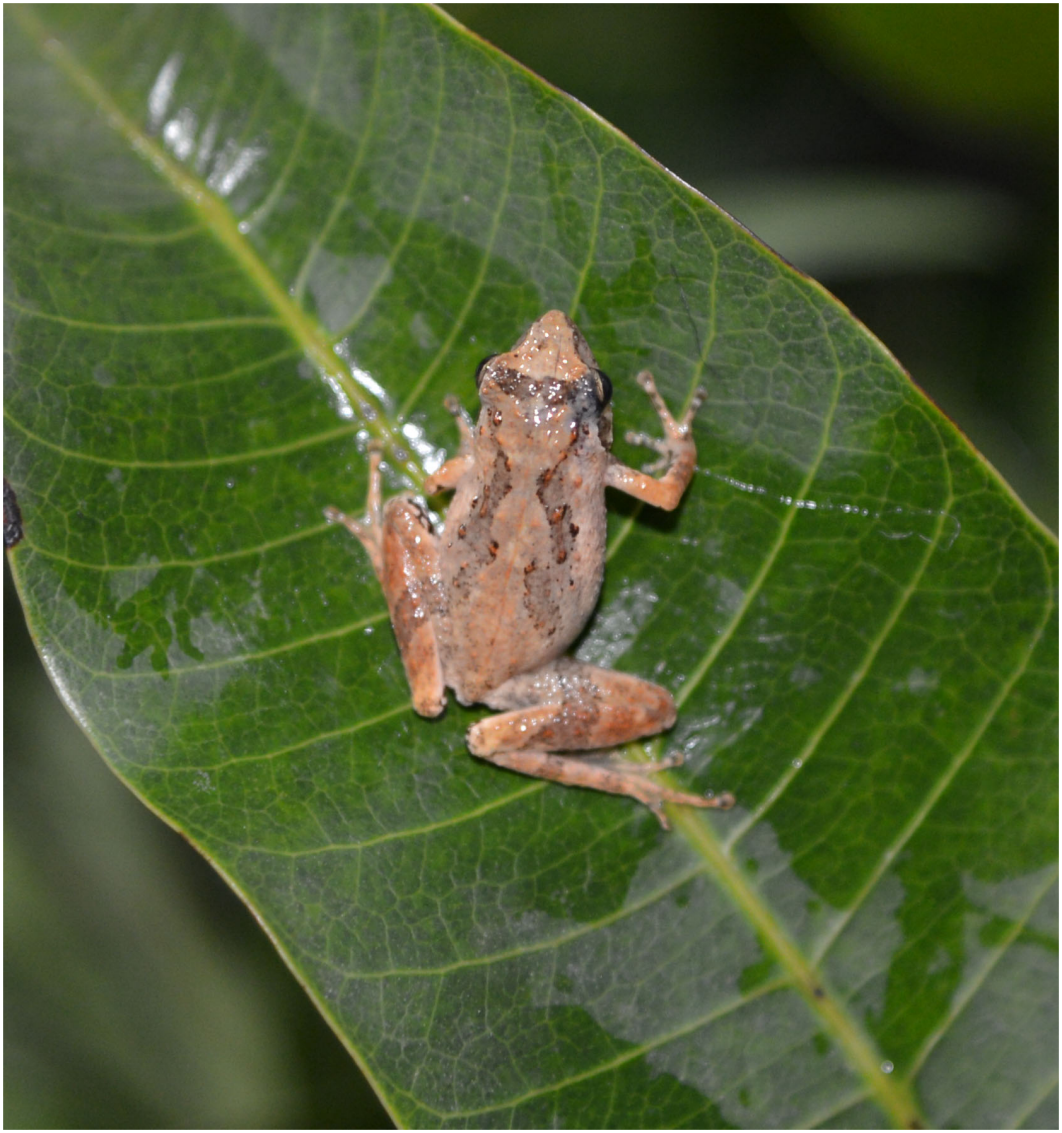
*Liuixalus shiwandashan* sp. nov., dorsal view. In life, male, from Mt. Shiwanda.

**Fig 3:**
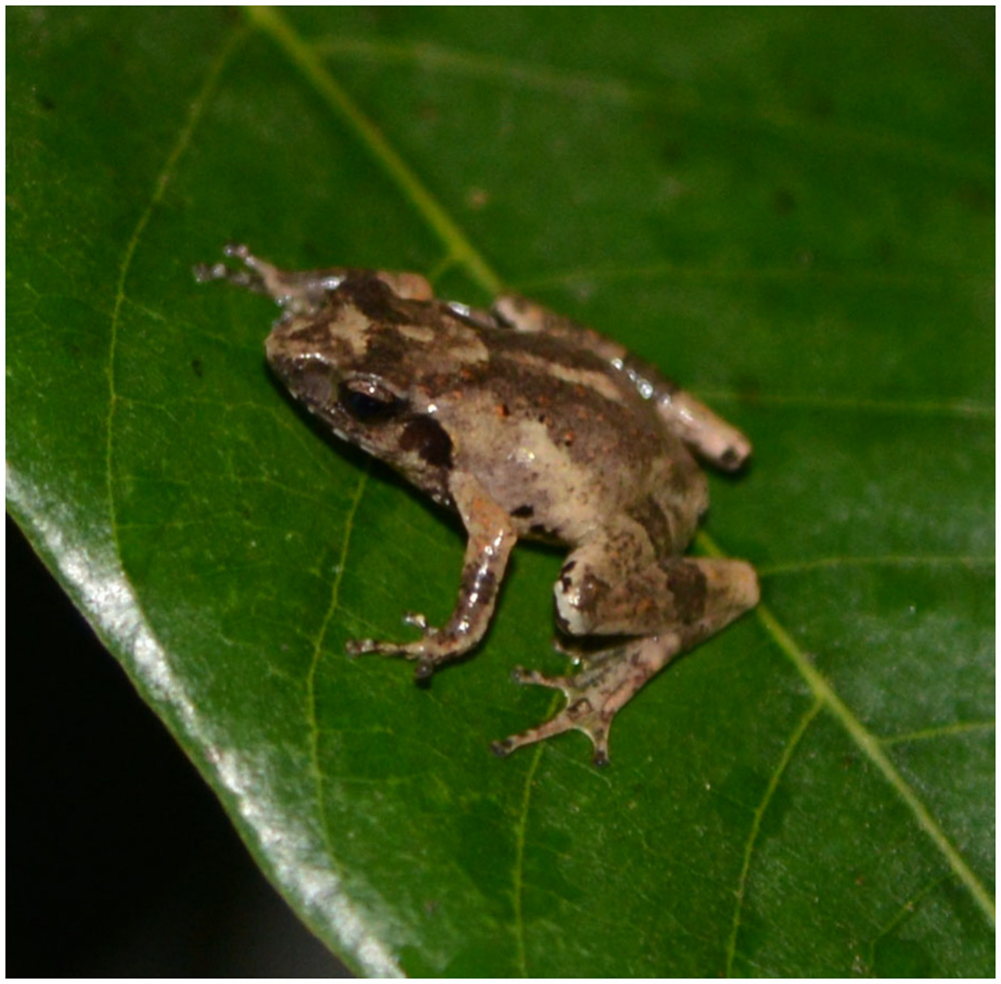
*Liuixalus shiwandashan* sp. nov., dorso-lateral view. In life, male, from Mt. Shiwanda.

CIB 101052, an adult male (Figs 2 and 3) from Mt. Shiwanda, Guangxi, China (21.72064°N 107.5427°E, elevation 937m a.s.l.), collected by Jia-Tang LI and Bo CAI on 24 April 2013.

#### Paratypes (12)

CIB 101053, CIB 101055–101059, CIB 101061, adult males, and CIB 101050, CIB 101051, CIB 101054, adult females, collected together with the holotype.

#### Etymology

The new species was named after the type locality, Mt. Shiwanda.

#### Diagnosis

*Liuixalus shiwandashan* **sp. nov.** is associated with *Liuixalus* in having a small body-size; head relatively broad; obtusely pointed snout; the right and left heel obviously overlap with each other; and a distinctly granulate “)(” pattern on the dorsum skin and upper eyelid.

*Liuixalus shiwandashan* **sp. nov.** is distinguished from all other species of *Liuixalus* by the following combination of morphological characters: 1) fingers with prominent dermal fringes; 2) flat tubercles on the ventral surface; 3) tibia-tarsal articulation reach (female) or extend beyond (male) nostril; 4) skin on dorsum smooth with sparse and compressed warts; 5) lateral side varicose in male; 6) nuptial pad covered by small whitish spines, present on the first and second fingers.

#### Description of the Holotype

Adult male (Figs 2 and 3), body length 17.14 mm. Further measurement provided in Table 2. Body slender; head longer than wide (HL/HW = 1.18); snout rounded, slightly protruding, projecting beyond jaw slightly; canthus rostral rounded, loreal region oblique and slightly concave; eye large, horizontally longer than distance from eye to nostril; tympanum distinct and medium sized, larger than half eye width; vomerine teeth absent, tongue large, oval, without papillae; dorsum skin smooth with a few, widely dispersed, inconspicuous tubercles; ventral skin verrucose.

**Table 2:** Measurements (in mm) of *Liuixalus shiwandashan* sp. nov. and *Liuixalus jinxiuensis* sp. nov.

	*Liuixalus shiwandashan* sp. nov.											*Liuixalus jinxiuensis* sp. nov.				
	Paratypes											Paratype				
	holotype	Males							females			holotype	males			females
	101052	101053	101055	101056	101061	101057	101058	101059	101050	101051	101054	200804109	200804107	200804108	200804110	101060
SVL	17.14	18.40	17.06	16.20	17.76	18.49	17.30	16.35	19.33	19.62	19.17	17.48	16.58	16.61	15.86	18.84
A-G	7.30	8.15	6.51	7.24	7.90	8.07	6.85	6.78	8.11	8.07	8.61	6.67	4.49	6.72	6.13	6.97
HL	6.78	7.51	7.00	7.63	7.96	7.65	7.36	7.51	7.11	6.92	7.07	5.81	6.41	6.61	6.28	6.63
HW	5.75	5.91	6.04	5.39	5.71	6.02	5.68	5.66	6.44	6.15	6.58	5.62	6.22	6.06	5.68	6.42
HD	3.41	4.82	3.92	3.77	3.99	4.10	4.62	3.71	3.58	3.96	4.50	3.31	3.16	4.11	3.39	4.03
UEW	1.13	1.05	1.25	1.06	1.42	1.26	1.38	1.20	1.44	1.30	1.35	1.09	1.27	1.14	1.22	1.34
IOD	3.37	4.01	3.82	3.34	3.64	4.07	4.67	3.88	4.38	3.67	4.79	2.62	3.17	2.85	2.78	3.30
ED	1.68	1.78	2.33	2.23	2.47	2.05	2.69	2.18	2.08	2.31	2.07	2.23	2.37	2.65	2.48	2.48
TD	1.40	1.09	1.20	1.00	1.93	1.21	1.21	1.34	1.41	1.27	1.27	0.90	0.96	1.09	0.70	1.09
ESL	2.51	3.49	3.03	2.76	2.96	2.68	3.07	2.35	3.04	3.15	2.83	2.81	2.93	2.67	2.76	3.55
TED	0.47	0.46	0.38	0.43	0.50	0.44	0.55	0.40	0.59	0.43	0.50	0.49	0.57	0.49	0.75	0.53
IND	2.06	1.92	2.00	1.78	1.91	1.86	2.02	1.75	2.25	2.31	2.14	2.21	2.19	2.03	2.27	2.71
END	1.28	1.52	1.09	1.37	1.25	1.25	1.24	1.48	1.36	1.52	1.58	1.29	1.43	1.34	1.25	1.47
FLL	8.38	7.38	7.62	6.93	7.35	8.51	6.87	7.53	9.48	8.73	8.05	7.34	7.13	7.29	7.15	7.82
FFL	1.00	1.32	1.16	1.07	1.25	1.13	1.22	1.19	1.77	1.36	1.17	1.50	1.55	1.06	1.31	1.26
TFL	2.93	2.81	2.86	2.81	3.47	3.26	2.98	2.68	3.34	3.09	3.44	1.77	1.70	1.69	1.60	1.89
FTD	0.64	0.60	0.64	0.45	0.49	0.51	0.58	0.63	0.54	0.53	0.72	0.51	0.72	0.57	0.40	0.70
Relative fingers length	1<2<4<3	1<2<4<3	1<2<4<3	1<2<4<3	1<2<4<3	1<2<4<3	1<2<4<3	1<2<4<3	1<2<4<3	1<2<4<3	1<2<4<3	1<2<4<3	1<2<4<3	1<2<4<3	1<2<4<3	1<2<4<3
Relative toes length	1<2<3 = 5<4	1<2<3 = 5<4	1<2<3 = 5<4	1<2<3 = 5<4	1<2<3 = 5<4	1<2<3 = 5<4	1<2<3 = 5<4	1<2<3 = 5<4	1<2<3 = 5<4	1<2<3 = 5<4	1<2<3 = 5<4	1<2<3 = 5<4	1<2<3 = 5<4	1<2<3 = 5<4	1<2<3 = 5<4	1<2<3 = 5<4

Forelimb long, length from tip of disk of finger III to axilla accounts for 48.77% of SVL. Finger slender, with strongly reduced interdigital web; dermal fringe prominent; relative lengths I < II < IV < III; tips rounded, enlarged; subarticular tubercles indistinct; inner metatarsal tubercle elliptical and prominent; outer metatarsal tubercle large but flat.

Hind-limbs long and slender; TBL 9.62 mm accounting for 56.13% of SVL; relative toes length I < II < III = V < IV; rounded discs on toes smaller than on fingers; webs between toes rudimentary; dermal fringe obvious; inner metatarsal tubercle flat and distinctively smaller than exterior metatarsal tubercle.

#### Coloration of holotype in life

Dorsal and lateral body yellowish brown; dorsum with brown “)(”-shape pattern from behind the head to the sacrum; puce subtriangular markings lying in the interorbital region. Dorsal part of limb yellowish and decorated with brown transverse bands. Throat, chest and belly immaculate yellowish white to white with sparse dark blotching; ventral surface of limbs transparent gray. Under jaw decorated with black stripe. Iris dark charcoal gray.

#### Coloration of holotype in preservative

In general, brightness reduces and tends to grayish-brown. Snout and dorsum brownish gray with brown pattern forming a “)(”. Ventral part of limbs and belly yellowish gray.

#### Variation

Variation in measurements given in Table 1. Females larger than males, SVL 19.2–19.6 mm in females (n = 3) and 16.2–18.5 mm in males (n = 9). Lateral tubercles of males more evident than for females. Individuals relatively uniform in body coloration.

#### Secondary sexual characters

Male internal single subgular vocal sac with a pair of slit-like vocal sac openings near corners of mouth; nuptial pads covered by small whitish spines present on dorsal side of the first finger and inner side of the second finger.

#### Comparison

*Liuixalus shiwandashan* **sp. nov.** differs from *L*. *hainanus* by having nuptial pads on both 1st and 2nd finger in males (only one nuptial pad in 1st finger for *L*. *hainanus*) and tibia-tarsal articulation reaching or beyond nostril (exceeds snout in *L*. *hainanus*). *Liuixalus shiwandashan* **sp. nov.** differs from *L*. *calcarius* by the absence of dark color on throat vocal sac (present in *L*. *calcarius*). *Liuixalus shiwandashan* **sp. nov.** differs from *L*. *ocellatus* by the presence of a prominent dermal fringe (absent in *L*. *ocellatus*) and tibia-tarsal articulation reaching or extending over nostril (reaches anterior eye in *L*. *ocellatus*). *Liuixalus shiwandashan* **sp. nov.** differs from *L*. *romeri* by presence of a prominent dermal fringe (absent in *L*. *romeri*).

#### Distribution

This species is currently known only from the type locality.

**Fig 4:**
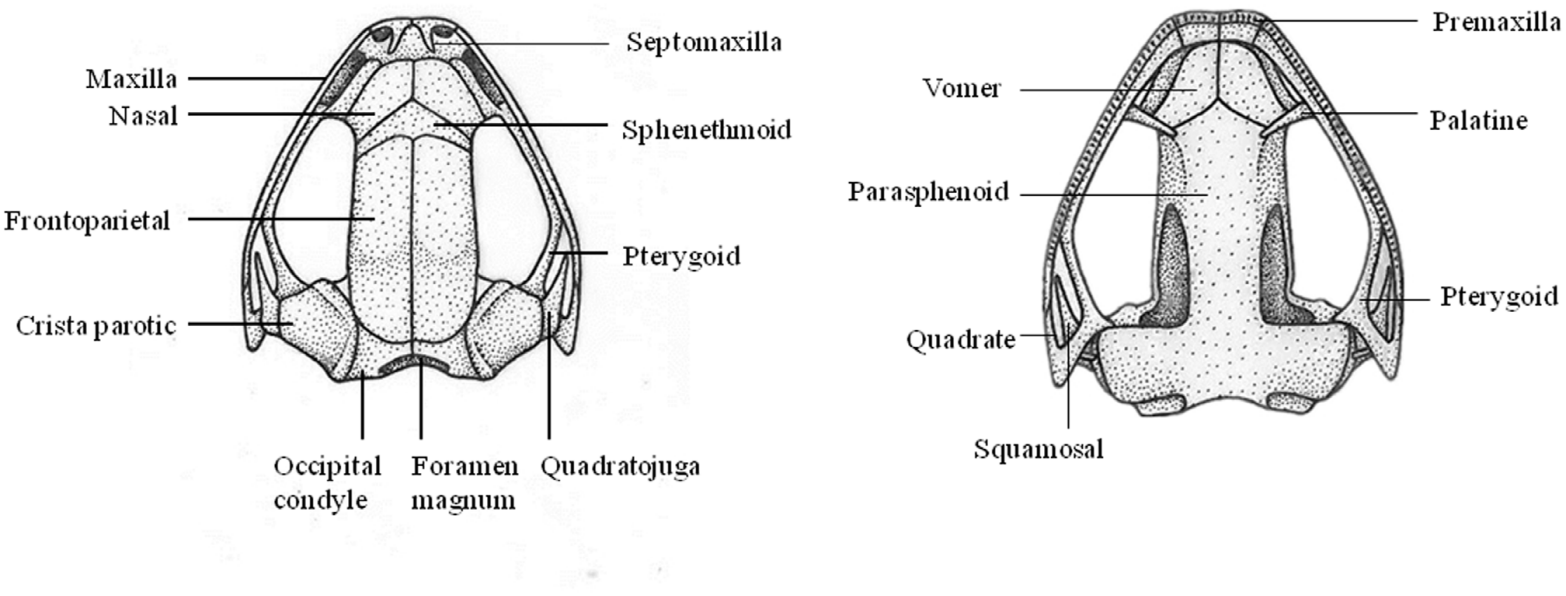
Skull of *Liuixalus shiwandashan* sp. nov. Left, dorsal view; right, ventral view.

The results of bioacoustic and osteological analyses, and the habitat were shown at Figs 4. 5 and 6.

**Fig 5:**
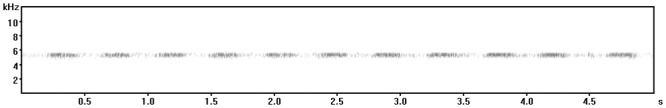
Spectrogram of calls series of *Liuixalus shiwandashan* sp. nov. The recording was high-pass filtered (above 5.5 kHz) to avoid high-frequence noise. Calls for figure spectrogram were created using 22005 Hz sampling frequency. Figure spectrogram was created using Hamming window, FFT-length 512 points, frame 100%, and overlap 75%.

**Fig 6:**
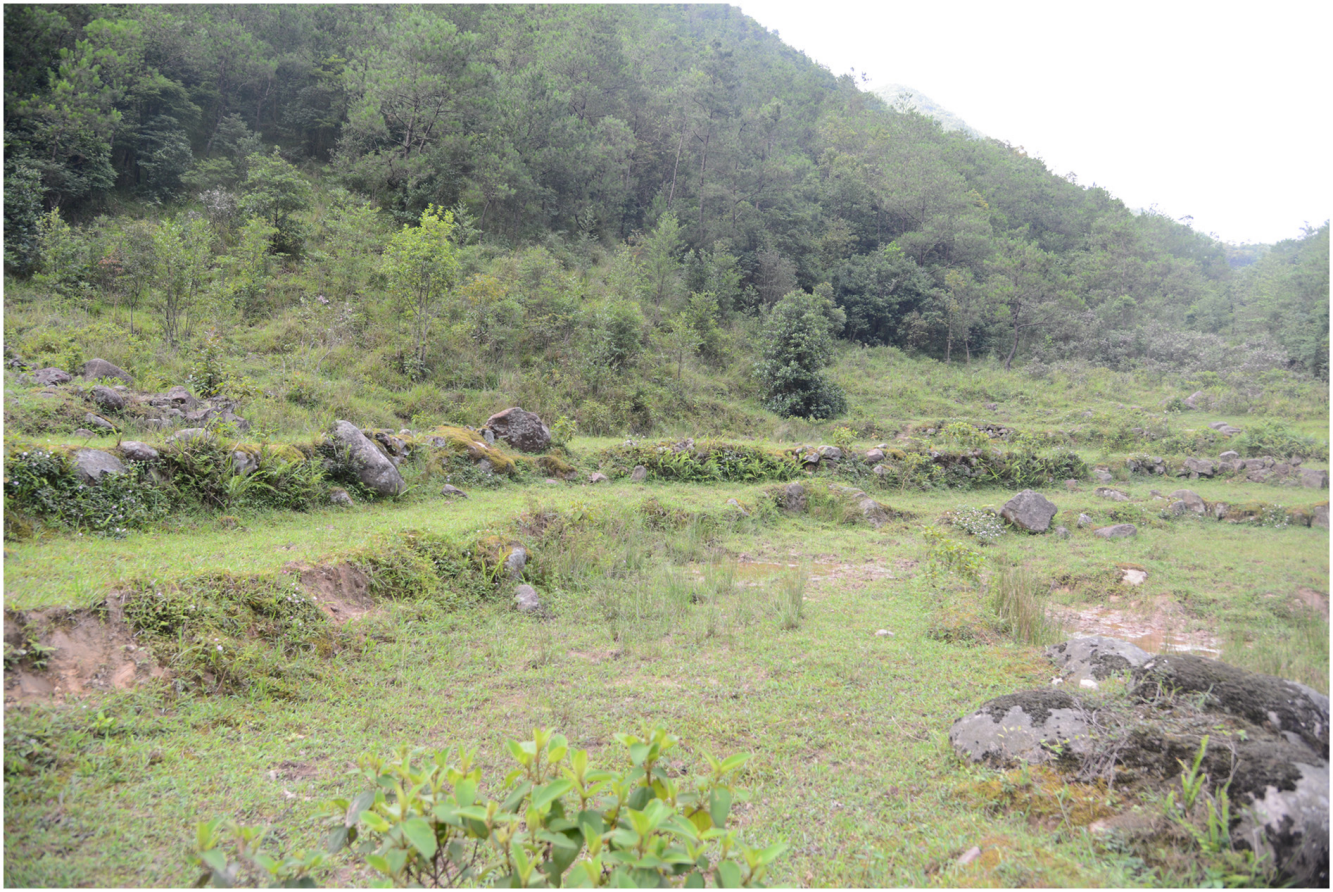
The habitat of *Liuixalus shiwandashan* sp. nov. in Mt. Shiwanda.

### *Liuixalus jinxiuensis* sp. nov.

(Li, Mo, Jiang, Xie & Jiang)

urn:lsid:zoobank.org:act:A0E3794E-1963-4DF2-BEED-2EB33D107688

#### Holotype

**Fig 7:**
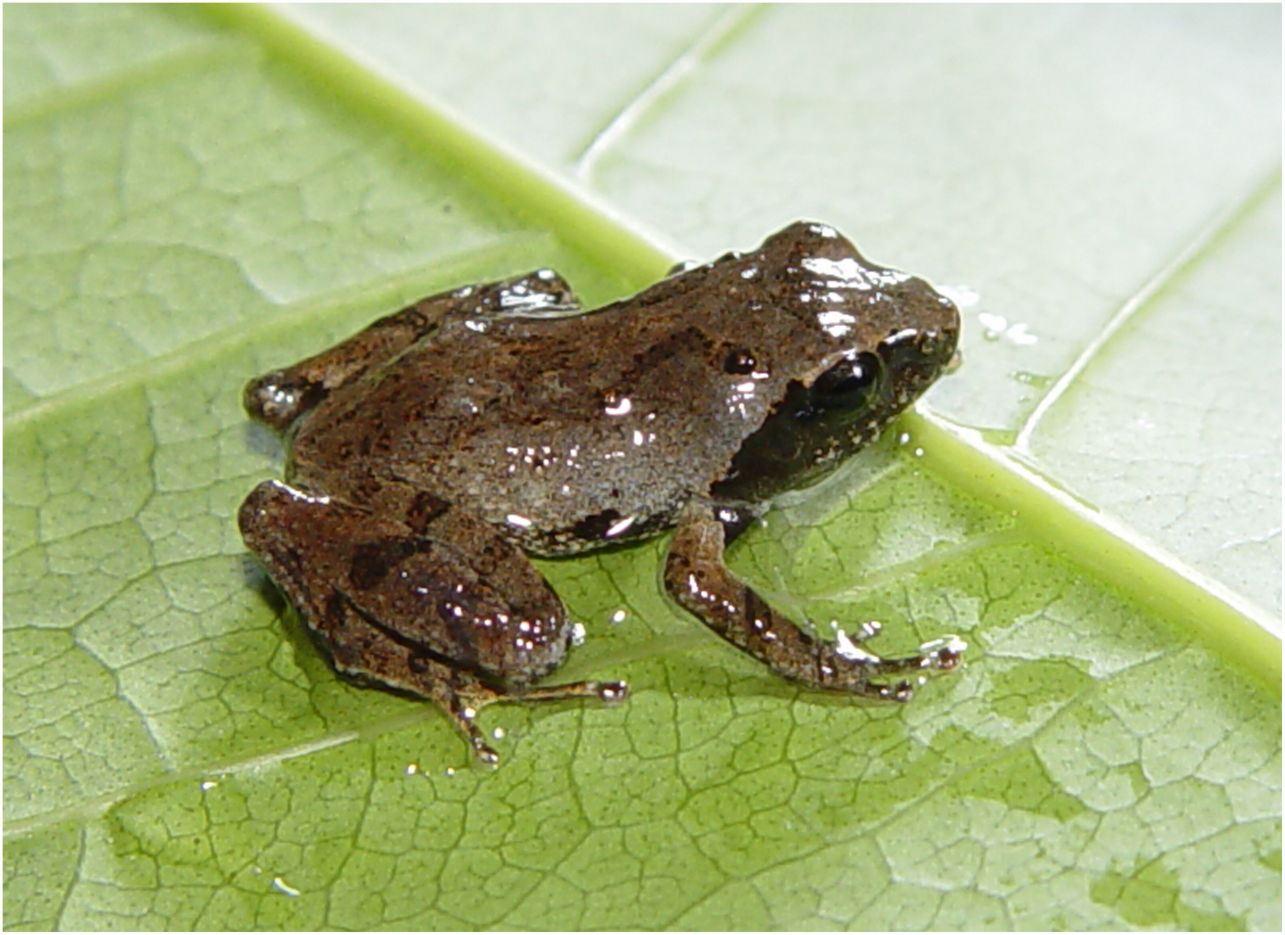
*Liuixalus jinxiuensis* sp. nov., dorso-lateral view. In life, from Jinxiu, Mt. Dayao.

**Fig 8:**
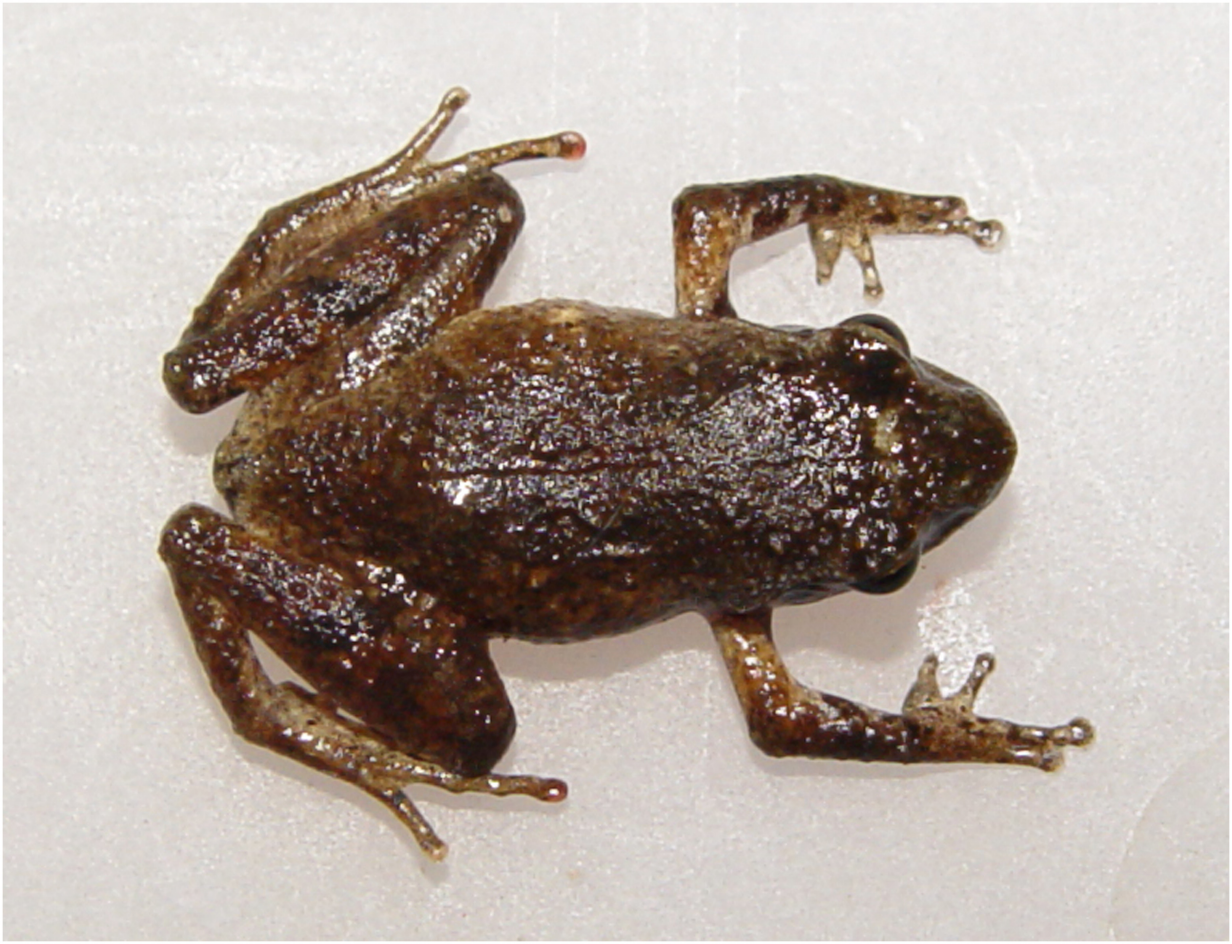
*Liuixalus jinxiuensis* sp. nov., dorsal view. In life, from Jinxiu, Mt. Dayao.

**Fig 9:**
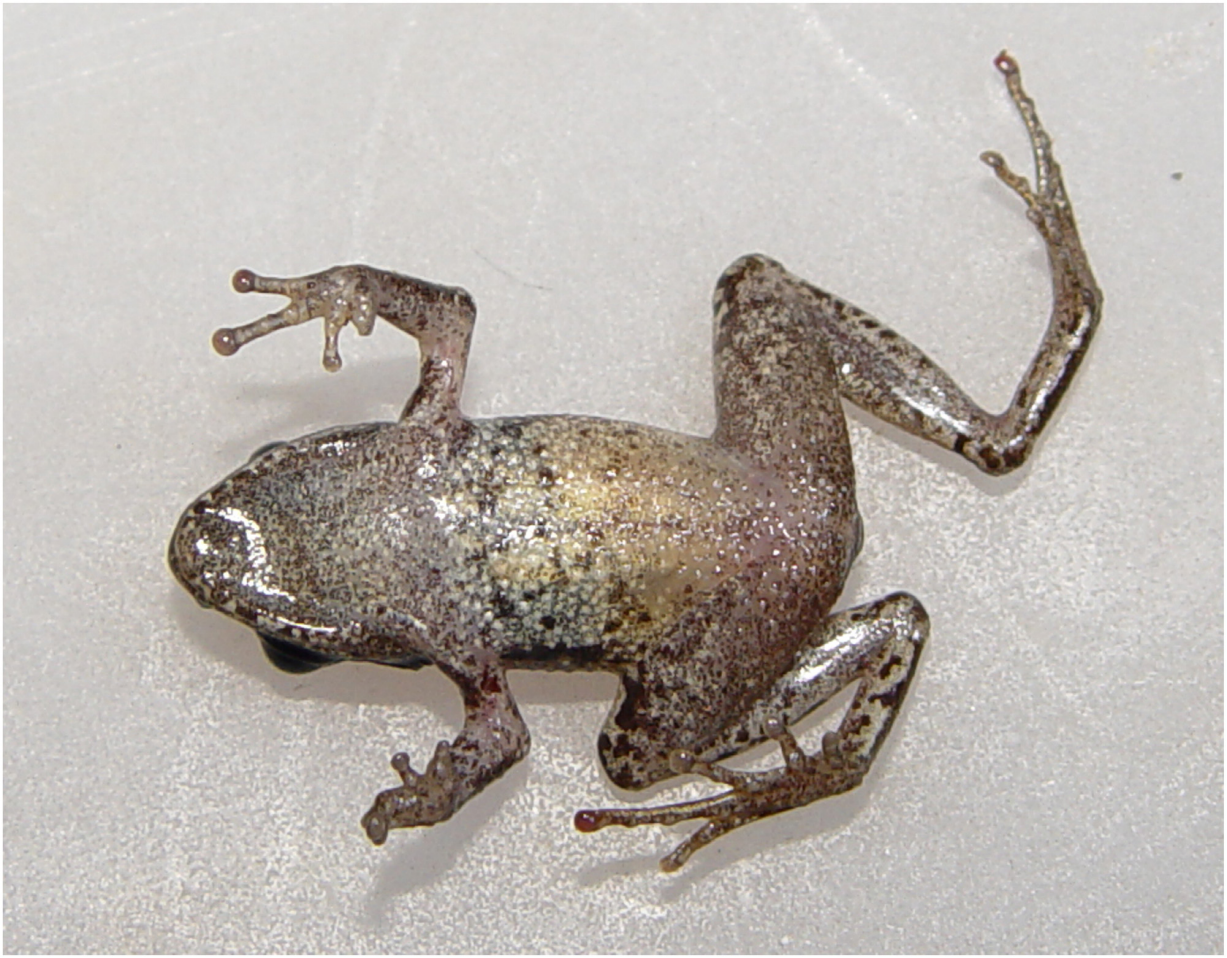
*Liuixalus jinxiuensis* sp. nov., ventral view. In life, from Jinxiu, Mt. Dayao.

GXNM200804109, an adult male (Figs 7, 8 and 9) from Mt. Dayao, Jinxiu, Guangxi, China (110°14.291′E 24°06.019′N 1163m), collected by Yunming MO and Shichu ZHOU, deposited in Guangxi Zhuang Autonomous Region Museum of Natural History.

#### Paratype

GXNM2000804107–108, GXNM2000804110, three adult males, collected together with the holotype; CIB101060, adult female from Mt. Dayao, Jinxiu, Guangxi, collected by Jia-Tang Li, deposited in Chengdu Institute of Biology, Chinese Academy of Sciences.

#### Etymology

The new species was named after the type locality, Jinxiu.

#### Diagnosis

*Liuixalus jinxiuensis* **sp. nov.** is assigned to the genus *Liuixalus* on the basis of the following characters: small body-size; head relatively broad; obtusely pointed snout; no vomerine teeth; tympanum distinct; the right and left heel obviously overlap with each other.

*Liuixalus jinxiuensis* **sp. nov.** is distinguished from all other species of *Liuixalus* by the following combination of morphological character: 1) large black plaque on the cephalic edge, under the eye (especially evident in living specimens); 2) sparse flat wart on the dorsal skin; 3) tibia-tarsal articulation reaching anterior eye; 4) light-colored nuptial pad present on the first and second fingers; and 5) internal single subgular vocal sac in males.

#### Description of the Holotype

Adult male (Figs 7, 8 and 9), body length 16.61 mm. Further measurement provided in Table 1. Body compact; head longer than wide (HL/HW = 1.09); snout slightly pointed in dorsal view; canthus rostral rounded and obvious, loreal region slightly concave; eye large, the horizontal of eye is less than eye to nostril distance; tympanum distinctly visible, rounded, less than half width of eye; vomerine teeth absent, tongue large and oval, distinctively notched posteriorly; skin on dorsum smooth with little and sparse tubercles; ventral skin non-uniformly verrucose.

Forelimb 7.29 mm, accounts for 43.89% of SVL. Finger slender, with strongly reduced interdigital web, no dermal fringe; relative finger length I < II < IV < III; finger discs rounded and expanded; subarticular tubercles indistinct; inner metatarsal tubercle elliptical and inconspicuous; outer metatarsal tubercle large but flat.

Hindlimbs relatively short, tibiotarsal articulation only reaching anterior eye when adpressed to the body; heels overlap when folded at right angle to body; relative toe length I < II < III = V < IV; rounded discs with ventral circummarginal groove; webs between toes rudimentary; dermal fringe missing; inner metatarsal tubercle flat and smaller than exterior metatarsal tubercle.

#### Coloration of holotype in life

Dorsal and lateral surface dark claybank to sepia, with sparse, dark, carbonarius ‘)(’-shaped tubercles; upper surface of forelimb same as dorsum with numerous dark-brown spots; ventral surface densely covered with hoary speckles and yellow asymmetrical spots mainly distributed on thorax-abdominal skin (Fig 9). Bilateral dark colored fusiform markings under eyes. Underjaw decorated with black stripe. Iris is celadon.

#### Coloration of holotype in preservative

Brightness reduces and tends to grayish-brown color. Bark tubercles fogged.

#### Variation and measurement of paratypes

Variation given in Table 1. Female SVL (18.84 mm) apparently larger than male (15.86–17.48 mm; n = 4).

#### Secondary sexual characters

An internal single subgular vocal sac is present in males. A pair of slit-like vocal sac openings occurs near the corners of the mouth. Nuptial pad present on dorsal side of the first finger and inner side of the second finger.

#### Comparison

Compared with all other congeners, *Liuixalus jinxiuensis* **sp. nov.** differs from *L*. *hainanus* by the nuptial pad occurring on both 1st and 2nd finger of males (only on 1st finger for *L*. *hainanus*) and tibia-tarsal articulation reaching anterior eye (exceeds snout in *L*. *hainanus*). *Liuixalus jinxiuensis* **sp. nov.** differs from *L*. *calcarius* by the absence of dark color on throat vocal sac (present in *L*. *calcarius*). *Liuixalus jinxiuensis* **sp. nov.** differs from *L*. *ocellatus* by the bilateral dark colored fusiform markings lying under eyes (small black dollar-spot on occiput of *L*. *ocellatus*), small, sparse tubercles on dorsal skin (large, dense tubercles in *L*. *ocellatus*), and ventral skin not uniformly verrucose (well-proportioned fine point on ventral skin in *L*. *ocellatus*). *Liuixalus jinxiuensis* **sp. nov.** differs from *L*. *romeri* by its large black plaque on the cephalic edge, under the eye. *Liuixalus jinxiuensis* **sp. nov.** differs from *Liuixalus shiwandashan* **sp. nov.** by absence of dermal fringe.

#### Distribution

This species is currently known only from the type locality.

**Fig 10:**
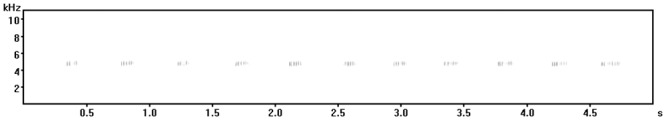
Spectrogram of calls series of *Liuixalus jinxiuensis* sp. nov. The recording was high-pass filtered (above 5.5 kHz) to avoid high-frequence noise. Calls for figure spectrogram were created using 22005 Hz sampling frequency. Figure spectrogram was created using Hamming window, FFT-length 512 points, frame 100%, and overlap 75%.

The result of bioacoustic analysis was shown at Fig 10.

## Discussion

We described *Liuixalus shiwandashan* **sp. nov.** and *Liuixalus jinxiuensis* **sp. nov.** using morphological, genealogical, bioacoustic and osteological analyses. Morphologically, both new species can be reliably distinguished from their congeners. Although the present genetic analyses were based on only one mitochondrial gene, the genetic difference between the two new species was of a comparative magnitude as other known *Liuixalus* species. We treated them as separate species based on the “biological species concept” [27–28].

Our matrilineal genealogy of *Liuixalus* depicted relationships among members of the group based on comprehensive sampling. Although we resolved six matrilines, relationships *intra se* did not always enjoy strong support.

Lineage A—*L*. *ocellatus*: Liu *et al*. [7] investigated the amphibians of Hainan Island and described *Philautus ocellatus* as a new species based on morphological characters. At 2009, Fei *et al*. assigned it to *Aquixalus* in *Fauna Sinica*. Based on molecular investigation, Li *et al*. [1] suggested the genus *Aquixalus* as a junior synonym of *Kurixalus*. Later, Li *et al*. [2] assigned *P*. *ocellatus* into *Liuixalus* based on molecular analysis.

Lineage B—*Liuixalus jinxiuensis* **sp.nov.**: In this study, molecular analysis showed a distinct clade related to this species. We suggested it to be a new species using extensive sampling and various analyses. Description was given above.

Lineage C—*L*. *calcarius*: Milto *et al*., 2013 and Nguyen *et al*., 2014 described a new species of *Liuixalus* from Cat Ba Island, Vietnam named *L*. *calcarius* and *L*. *catbaensis*, respectively. We suggested that *L*. *catbaensis* was a junior synonym of *L*. *calcarius* by priority of date of publication, which was consistent with the suggestion of Frost (2014). Regardless, the occurrence of the species demarked the first record of *Liuixalus* outside China.

Lineage D—*L*. *hainanus*: Liu *et al*. (2004) described *Philautus hainanus* as a new species based on morphological evidence: tibiotarsal articulation over the tip of snout, 2~3 dark cross-bands on back of lower arm, beige ellipse speckle on the middle part of the body back in life, and yellow-white on abdomen. Then this species was then assigned to *Liuixalus* by Li *et al*. [2].

Lineage E–*Liuixalus shiwandashan* **sp. nov.**: Molecular analysis showed a distinct clade related to this species. Using extensive sampling and various analyses, we suggested it to be a new species. Description was given above.

Lineage F—*L*. *romeri*: *Liuixalus romeri* was firstly described as *Philautus romeri* by Smith in 1953 from Lamma Island, Hong Kong, China. Subsequently, this species was transferred among several genera [29–30][1]. Due to the presence of a tadpole, *Philautus romeri* was tentatively assigned to *Chirixalus* [29]. Wilkinson *et al*. [14] suggested that *P*. *romeri* may be a putative member of *Kurixalus*, pending further study of the type specimens and specimens in the field. Frost *et al*. [30] moved this species to the genus *Chiromantis*, pending resolution of its phylogenetic position. Li *et al*. [1] assigned it to *Liuixalus* based on both morphological and molecular evidence.

**Fig 11:**
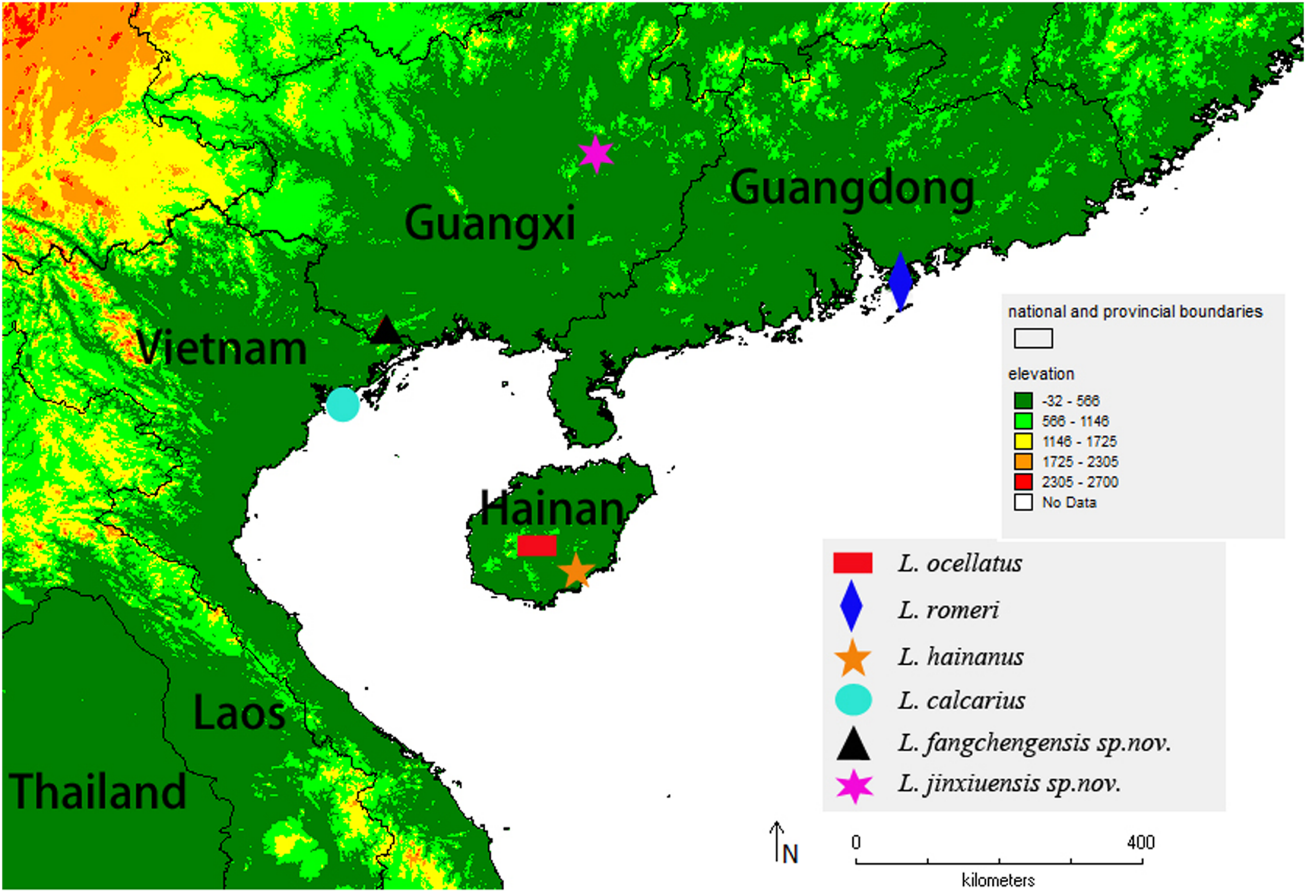
Collection sites of the species in this study.

The localities of these species were marked in the map (Fig 11), and they were well separated geographically. The close relationships among *Liuixalus* suggested an ancient link between Hainan, Guangxi, Hong Kong and Vietnam. According to the geological evidence, the separation between Hainan Island and mainland China occurred at Early Cretaceous Epoch [31–32]. The separation accelerated the speciation within the genus *Liuixalus*.
